# Does Air Enter the Uterine Veins during Labor, or Shortly after, and Cause Death?

**Published:** 1858-03

**Authors:** J. P. DeBruler

**Affiliations:** Rockport, Ind.


ARTICLE II.

### DOES AIR ENTER THE UTERINE VEINS DURING LABOR, OR SHORTLY AFTER, AND CAUSE DEATH?

BY J. P. DE BRULER, M.D., ROCKPORT, IND.

As this is to some extent an open question, and at the same time a grave one, I submit the following cases which came under my observation, thinking they may have some bearing upon the subject. I regret that I am able to furnish nothing more than approximative testimony, no post mortem examination having been made in any of the cases.

CASE 1.—I was called to see Mrs. B., aged 36, of stout, muscular frame, who had usually enjoyed excellent health, on the night of April 21st, 1852, in labor with her first child. She had been complaining some three days, and during this time had been bled and moderately purged. I found her with regular pains, though rather feeble, and the os uteri dilated to the size of a dollar, and the membranes ruptured. My patient being cheerful, and feeling comparatively well, I left her for the night in care of her midwife. Called again in the morning about nine o'clock, found some progress, os more dilated, but not yet sufficiently so to admit of the passage of the child's head. At eleven o'clock, the pains were forcible, the vertex pressed upon the os uteri, which was yet thick, firm and unyielding. I now resolved to bleed to the point of obtaining the necessary relaxation, but, upon examining the state of the circulation, I found blood-letting totally inadmissable; indeed, I could not count her pulse, so rapid was the circulation. This very much astonished me, as up to that time I had not noticed any flagging. I now observed, for the first time, that she breathed rapidly, and had a wild, anxious countenance. My attention was of course anxiously directed to the progress of her labor, hoping that delivery might avert what seemed to me an impending fate. The very next pain astonished me, by bringing away a *gush of air from the uterus.* The report was loud, and I could distinctly feel the impulse. The egress of air recurred at almost every pain until the child was born, which occurred in about an hour. The report, I said, was loud—it was truly so, and annoyed her and her friends exceedingly, they supposing it came from the rectum. I did not undeceive them, for fear of creating alarm. The child presented every appearance of having been dead several days. Large patches of cuticle were filled with foul and very offensive gases, the body being much distended.

I saw with pain that her respiration became more and more embarrassed, and her countenance wild and anxious. She was placed in bed, and a cordial and opiate administered. I then ran out myself to call in my friend, Dr. Crooks, who I knew to be near by. We returned immediately, but she did not live more than five minutes. There was no hemorrhage, and to be certain that there was none concealed, I passed my hand into the uterus, which was found firmly contracted, and contained a small clot of blood, probably two or three ounces.

Case 2.—In 1842, I was called to see Mrs. R., in a premature labor. She supposed herself in the sixth month of her pregnancy. I found her with active bearing-down pains, the os well dilated, and the membranes tense. While engaged in ascertaining the presentation, a pain came on which ruptured the membranes, a considerable quantity of water was discharged, and the head came down to the inferior strait. Just then she cried out, "Raise me up, or I will faint." Some person near her head elevated it a little, when she became slightly convulsed, and died in less than one minute. Thinking that it was only syncope, I placed my finger upon the pulse, and found the artery pulsating with considerable firmness, which it continued to do for some moments after she ceased to breathe.

Case 3.—Communicated by my partner, Dr. Crooks.—About ten o'clock P.M., of the 21st ultimo, called to see Mrs. P., aged 27; found her in labor with her first child; progressing naturally enough, with only the slight tardiness common to persons of her age. A healthy child was the result at one o'clock next morning. There seemed nothing uncommon during the labor, only a rather unusual intolerance to pain. Immediately after delivery, syncope, difficult respiration, flagging of the circulation, and most intolerable after-pains, supervened. Notwithstanding the after-pains, I suspected hemorrhage, but, upon examination, found none of consequence. The uterus was firmly contracted. Gave opiates, and stayed with her two hours; left, thinking her better, but not without apprehension that something was wrong. About two o'clock, a renewal of the unpleasant symptoms occurred. Being absent from my office and engaged, I did not see her until nine o'clock P. M., when I found her much in the same condition above described; except, instead of after-pains, she had *universal* pain—thighs, bowels, region of the heart, and particularly severe in the shoulders, and with more frequent and alarming syncope. The pains were transitory—passing from place to place instantaneously. I again examined the condition of the womb, and found it firmly contracted, with no loss of blood since I left in the morning. She continued to grow worse, and died at ten o'clock. I will add, that when the placenta was expelled, I distinctly noticed a gush of air from the vagina, or at least that was the impression made upon my mind at the time, but it occurring but once, I cannot speak as positively to the fact as I could desire.

The above cases are not introduced for the purpose of proving that air ever enters the veins in labor, for it is manifest that nothing short of a post mortem proof could be positively relied on, but they are reported more for the purpose of attracting the attention of the profession to the subject. I now think if ever I am unfortunate enough to meet with another such case, I will try with all the influence I can bring to bear upon the subject to procure a post mortem examination. The cases all occurred in families who could not be induced to submit their friends to such an examination—a feeling that is too general in this country for the benefit of our profession, and one that every physician should exert himself to remove.

All of the cases may not have died from the cause above indicated. Indeed, none of them may; yet it occurs to me, that the symptoms and result, to some extent, favor the hypothesis. Especially does this seem to be true with case No. 1. She was a stout, robust woman, apparently enjoying excellent health up to her confinement, was able, during all her labor up to the last hour, to walk about her room, which she persisted in doing, saying that it rested her. The failure of her strength was sudden, and to me unexpected. Her death could not be accounted for upon (to me) any other known principle. It could not have been from hemorrhage, for there was almost none, neither external nor concealed. And then we have the fact, that air or gases were in the uterus for some hours before delivery. About this I cannot be mistaken, for I both felt and heard it, at almost every pain, for an hour or more. Is it not reasonable that air could be forced into patulent uterine veins during active contraction of the organ?

Upon case No. 2 I have no comments to make, only, that its suddenness made a strong impression upon me at the time. She had not, so far as I could ascertain, previously suffered from any organic disease of the heart, nor was she known to suffer from any disease at all. There had been some hemorrhage, but certainly not enough to cut any material figure in the case. Indeed, up to my arrival, no person supposed her in any danger whatever, and yet I do not think I had been to her bed two minutes before she died.

Case No. 3 enjoyed her usual good health up to her confinement; only that she had suffered from chill and fever a few weeks previously, which, however, did not reduce her much. Her death appears wholly unaccountable upon any other hypothesis than the one under consideration.

